# Endoscopic findings in children with stridor.

**DOI:** 10.1016/S1808-8694(15)31021-1

**Published:** 2015-10-19

**Authors:** Regina H.G. Martins, Norimar H. Dias, Emanuel C. Castilho, Sérgio H.K. Trindade

**Affiliations:** a Doctor, Assistant Professor, Faculty of the Otorhinolaryngology Department at the Paulista State University (Unesp), Botucatu campus.; b Master on Surgery, Assistant Physician at the Otorhinolaryngology Department, Paulista State University (Unesp), Botucatu campus.; c Doctor on Surgery, Assistant Physician at the Otorhinolaryngology Department, Paulista State University (Unesp), Botucatu campus.; d Physician, ENT specialist, ex-resident at the Otorhinolaryngology Department, Paulista State University (Unesp), Botucatu campus. Ophthalmology, Otorhinolaryngology and Head & Neck Surgery Departments at the Paulista State University (Unesp), Medical School, Botucatu campus.

**Keywords:** Children, Endoscopy, Stridor

## Abstract

Congenital and acquired airway diseases are responsible for upper respiratory distress and stridor in children. In neonatal intensive care units, we have seen increased survival in premature babies, but also a high incidence of airway complications related to intubation, which present as stridor.

**Aim:**

To review endoscopic findings in children with stridor.

**Study design:**

a cross-sectional cohort study.

**Methods:**

A retrospective analysis was done of 55 cases of children with stridor who underwent endoscopic exams, between January 1997 and December 2003.

**Results:**

69% were aged below one year. The main indications for endoscopy were post-extubation stridor (63.63%) and evaluation of neonatal stridor (21.82%). Many associated diseases were seen, including lung diseases (60%), neurological condition (45.4%), and GERD (40%). The main endoscopic findings and indications for tracheotomy were subglottic stenosis (27.27%) and airway inflammatory process (21.82%) occurring in children under five years old. Congenital disorders were more frequent in children under age one year.

**Conclusion:**

Neonatal stridor has many causes; those related to tracheal intubation are more frequent in hospitals that treat more complex diseases. Pediatricians and otorhinolaryngologists should know the main causes of stridor and perform detailed clinical evaluations to determine case severity. The endoscopic examination, must be meticulous.

## INTRODUCTION

Many congenital or acquired diseases may cause upper airway respiratory distress in children; stridor is the most common symptom in such cases. Stridor may be defined as the presence of noisy respiration resulting from the turbulent passage of air through a narrowed airway. Depending on its location, stridor may be inspiratory (pharynx or supraglottic), biphasic (glottic or infraglottic) or expiratory (trachea and lower airways).^1^

In newborn and lactating children, congenital airway anomalies are the most frequent cause of stridor, of which laryngomalacia is the main cause.^2^, ^3^, ^4^, ^5^ Other congenital malformations of the larynx that cause stridor include atresia, laryngeal membrane, cysts, infraglottic stenosis, laryngotracheal fissure, hemangiomas, laryngocele and vocal cord palsy.^1^, ^2^, ^3^

Technological developments in neonatal intensive care units have resulted in increased survival rates of extreme premature children that require ventilatory support. At the same time there has been an increase in the rate of airway injuries cause by tracheal intubation, including granulomas, laryngeal and tracheal stenosis. These last two conditions have become the main acquired causes of stridor in infancy.^6^, ^7^ According to Holinger,^2^ mild circumferential edema in the infraglottic region of a child drastically reduces the airway lumen due to the small diameter of the larynx in children, which results in stridor. Predisposing factor for intubation trauma include: inadequate diameter of the tracheal tube, superficial sedation resulting in excessive tube movement, traumatic and prolonged intubation, local or systemic infection, excessively high pressure in the tube cuff, hemodynamic disorders, prematurity, mechanical ventilation, and gastroesophageal reflux disease (GERD). Other causes of stridor in infancy include foreign body aspiration, vocal cord palsy, acute laryngitis, laryngocele, cysts and laryngeal papillomatosis.^8^, ^9^, ^10^, ^11^, ^12^, ^13^, ^14^

Prompt medical attention is essential to establish the severity of cases with stridor and define the degree of respiratory distress. In most cases detailed airway endoscopy will be required not only for the diagnosis but also to treat the condition. A flexible nasopharyngolaryngoscope or direct laryngoscopy and tracheobronchoscopy may be chosen. Flexible endoscopy does not require anesthesia and allows the examination of the nose, the pharynx, the supra and infraglottic regions and laryngeal dynamics. Lack of child collaboration and the high rate of synchronous airway lesions, particularly in congenital disease cases, preclude this method; in these situations direct laryngoscopy under general anesthesia is preferred in specific cases, allowing a more detailed assessment of the infraglottic and the tracheobronchial region.

The aim of this study was to conduct a retrospective analysis of endoscopy results (direct laryngoscopy direct and rigid tracheobronchoscopy) in children with stridor.

## CASES AND METHODS

Approval was obtained from the Research Ethics Committee for human studies, following which a retrospective study was made of the medical files of every child undergoing rigid upper airway endoscopy (direct laryngoscopy and rigid tracheobronchoscopy) under general anesthesia, due to respiratory distress and stridor, between January 1997 and December 2003, at the Hospital das Clínicas (Clinical Hospital) of the Faculdade de Medicina de Botucatu (Botucatu Medical School). Children included in this study had been admitted to the pediatric ward or the neonatal ICU due to severe respiratory distress and had not undergone flexible nasofibroscopy. At our unit this method is used only in mild or sporadic cases and children that cooperate. Children undergoing laryngoscopy for phonosurgery were excluded from the study.

Data obtained from the medical files included identification (gender and age), indication and results of endoscopy, co-morbidity and indications for tracheotomy. Results were presented descriptively and in percentages, and confronted with medical literature.

In our routine for endoscopy, anesthesia is initially superficial to allow an assessment of vocal cord mobility and laryngeal structures during breathing, allowing the detection of possible airway collapse in cases of laryngomalacia and tracheomalacia. Anesthesia is then deepened to allow the introduction of a rigid lens to reach distal airways. The exam is considered normal if airways are unobstructed, vocal cords are mobile, and there is no inflammation, no organic lesions, and no structural laryngeal or tracheal collapse during breathing.

## RESULTS

During the study period 73 children underwent airway endoscopy (direct laryngoscopy and rigid tracheobronchoscopy), however 18 medical files were incomplete and were excluded, resulting in a study group of 55 children.

**Gender:** There were 32 male children (58.2%) and 23 female children (41.8%).

**Age group:** Age varied from 2 days to 8 years. 69% were under age 1 year (Table 1). Three children were over five years.

**Table 1:** Distribution of children with stridor undergoing endoscopy, in age groups.

Age range	N	%
0 to 28 days	15	27.2
29 days to 1 year	23	41.8
1 to 5 years	14	25.5
> 5 years	3	5.5

Total	55	100.0

**Indication for endoscopy:** The main indications for endoscopy are shown on Table 2. We highlight stridor with post-extubation respiratory distress (n-35; 63.63%) and the assessment of neonatal stridor of unknown causes (n-12; 21.82%). Other indications included: recurring crises of cyanosis and stridor, suspected foreign body aspiration, progressive stridor-associated dysphonia, stridor following caustic soda ingestion, and stridor in children with bullous epidermolysis.

**Table 2:** Distribution of children relative to the indications for endoscopy.

Indications	N	%
Post-extubation stridor	35	63.63
Assessment of post-natal stridor of undetermined cause	12	21.82
Recurrent cyanosis and stridor attacks	3	5.45
Suspicion of foreign body aspiration	2	3.64
Stridor-related progressive dysphonia	1	1.82
Stridor after ingestion of caustic soda	1	1.82
Stridor in children with epidermolysis bullosa	1	1.82

Total	55	100.00

**Co-morbidity:** Stridor-associated lung diseases were detected in 60% of children, of which pneumonia was the most frequent. Neurological diseases were also significant, around 45.4% of cases, including seizures, non-progressive chronic encephalopathy, CNS malformation, meningitis, intracranial hypertension and hemorrhage, and the Guillan-Barré syndrome. Other conditions included gastroesophageal reflux disease (GERD) in children with stridor, which compounded about 40% of cases (n-22). An important point is that the diagnosis of GERD was based on noting regurgitation, which was confirmed by exams such and/or pH measurement. Other co-morbidities included prematurity, genetic syndromes and heart pathology (Table 3).

**Table 3:** Reported co-morbidity in 55 children with stridor undergoing endoscopy.

Comorbidities	N	%
Pulmonary	33	60.0
Neurologic	25	45.4
FLR	22	40.0
Prematurity	15	27.3
Genetic syndromes	13	23.63
Cardiac	12	21.8

**Endoscopy results and corresponding age groups:** The most frequent endoscopic findings were infraglottic stenosis (n-15; 27.27%) and laryngeal and/or tracheal inflammation (n-12; 21.82%), mostly in children aged under 5 years. Congenital diseases of the larynx and trachea causing stridor during the first year of life included bilateral vocal cord palsy (n-7), laryngomalacia (n-6), vascular tracheal compression (n-2), laryngeal cyst (n-1) and the redundant membranous portion of the trachea (n-1). Other less frequent diagnoses are listed on Table 4. Nine children (16.36%) had normal endoscopy.

**Table 4:** Results of endoscopy and age groups.

	Age range				
Results	0-28 days	28 days to 1 year	1 to 5 years	> 5 years	Total N(%)
Subglottal stenosis	0	3	12	0	15 (27.27)
Inflammatory process in the larynx and/or trachea	2	8	2	0	12 (21.82)
Bilateral vocal fold paralysis	3	3	0	1	7 (12.72)
Laryngomalacia	0	6	0	0	6 (10.90)
Extrinsic vascular tracheal compression	2	0	0	0	2 (3.64)
Subglottal granuloma	1	0	0	0	1 (1.82)
Congenital laryngeal cyst	1	0	0	0	1 (1.82)
Redundant tracheal membranous portion	1	0	0	0	1 (1.82)
Pierre Robin Syndrome	1	0	0	0	1 (1.82)
Normal exam	4	3	0	2	9 (16.36)

Total	15	23	14	3	55 (100.00)

**Indications for tracheotomy:** Of 55 children with stridor undergoing endoscopy, 41 required a tracheotomy (74.55%), the main indications being infraglottic stenosis (n-15) and secondary airway inflammation due to tracheal intubation (n-11). All children with vocal cord palsy and two of those with laryngomalacia also required tracheotomy. Other indications are listed on Table 5.

**Table 5:** Results of endoscopy and indications for tracheotomy.

Result	N (%)	Tracheotomy N (%)
Subglottal stenosis	15 (27.27)	15 (27.27)
Inflammatory process in the larynx and/or trachea	12 (21.82)	11(20.00)
Bilateral paralysis of vocal folds	7 (12.72)	7 (12.72)
Laryngomalacia	6 (10.91)	2 (3.64)
Extrinsic vascular trachea compression	2 (3.64)	2 (3.64)
Subglottal granuloma	1(1.82)	1(1.82)
Congenital laryngeal cyst	1(1.82)	1(1.82)
Redundant tracheal membranous portion	1(1.82)	1(1.82)
Epiglottis-malformation-related Pierre-Robin syndrome	1(1.82)	1 (1.82)
Normal exam	9(16.36)	0 (0.0)

## DISCUSSION

We observed that 70% of the children in our study were aged below 1 year, showing that stridor and respiratory distress are more frequent in this age group, as also reported by other authors.^2^, ^9^, ^14^ Respiratory distress with stridor in infancy is a dramatic situation that has a variety of causes. The medical assessment should include a first evaluation of respiratory involvement looking at the child's posture in the hospital bed, the presence of inspiratory and/or expiratory noises, nasal flapping, tachypnea, tachycardia, changes in the inspiratory and expiratory time ratio, cyanosis, pallor, sweating, chest indrawing, sleep loss and the feeding pattern.^1^, ^13^

Inclusion criteria were those children admitted to hospital that had stridor and that had undergone upper airway direct laryngoscopy and rigid tracheobroncoscopy under general anesthesia. These were severe cases that required more detailed exams. The study was done in a reference hospital for high complexity diseases. As such we expected a high rate of complications resulting from tracheal intubation, such as stenosis and inflammation of the mucosa, which are the main causes of tracheotomy. These are also the main causes of respiratory distress in infancy, and are directly related with laryngotracheal stenosis.^9^, ^13^, ^14^ According to Rocha et al.,^15^ when there is prolonged intubation, such as in children victims of cranial trauma, tracheotomy should be preferred to intubation, due to the lower risk of complications.

Benjamin^16^ reviewed extensively the intubation-related risk factors, highlighting laryngeal anomalies, the duration of intubation, the number of attempts to intubate, tube movement, gastroesophageal reflux, local acute infectious disease, systemic hemodynamic disturbances and inappropriate tube diameter. In previous papers we reviewed the main intubation-related airway complications, which included epithelial damage caused by balloon pressure on the mucosa,^17^, ^18^ and laryngeal injury in intubated patients that cause hoarseness, such as hematomas, laceration, granulomas, and anterior and posterior commissure synechiae.^18^, ^19^

Infraglottic stenosis and airway inflammation was seen at a higher rate in children under age 5 years in our study, probably due to the reduced airway diameter. Congenital laryngeal and tracheal lesions were more relevant in children aged below 1 year. These conditions included laryngomalacia, congenital laryngeal palsy, vascular compression of the trachea, laryngeal cysts and the redundant membranous portion of the trachea. These anomalies cause symptoms in the first days of life, explaining endoscopy at this age to clarify the etiology of stridor. Many of these cause severe symptoms requiring tracheotomy, as we saw in our study.

Medical literature suggests that laryngomalacia is the main cause of congenital stridor in infancy.^1^, ^2^, ^4^, ^5^ However, our study suggest that intubation-related laryngotracheal injury is more relevant. This was explained by the fact that children undergoing flexible nasofibroscopy were excluded from this study. This method is less invasive and assesses laryngeal dynamics more “naturally”; it is considered the gold standard in the diagnosis of laryngomalacia.^5^, ^20^ In severe cases, which were the cases in our study, direct laryngoscopy with rigid tracheobroncoscopy is considered the best method to investigate severe stridor in neonates and children, particularly when congenital or synchronic lesions are suspected.^4^, ^5^ Tracheotomy was indicated in two of the children in our study, an uncommon procedure for this anomaly, as it tends to progress favourably in months.^21^

Stridor may also be found in children with genetic syndromes that include upper airway malformation. In this group we highlight the Pierre Robin syndrome, where retrognatism compromises airway permeability due to the posterior position of the base of the tongue.^22^, ^23^ The child that presented this anomaly in our study also had epiglottic cartilage hypoplasia; airway permeability was obtained by a forward tongue movement and fixation, not requiring tracheotomy.

In many cases stridor in infancy is associated with pulmonary and neurological diseases, as seen respectively in 60% and 45.4% of children assessed. In this case airway endoscopy may be normal, as we saw in 9 children.

The association of stridor and GERD was also relevant, as highlighted by other authors, and may lead to the development of laryngospasm and infraglottic stenosis.^24^, ^25^, ^26^ There is a higher incidence of gastroesophageal reflux in children with laryngomalacia; GERD is considered a predisposing factor for the development of laryngomalacia.^26^

## CONCLUSION

Stridor in children is a frequent finding, and may reflect a variety of diseases. In university hospitals, where more complex diseases are usually seen, tracheal intubation-related complications are the main causes of stridor in children. ENT specialists and pediatricians should recognize the main causes of stridor and carefully analyze each case to establish severity. Endoscopy, when indicated, should be detailed and carefully done.
